# Real‐Time Empirical Risk Assessment From Recurrent Coastal Sewage Plumes

**DOI:** 10.1029/2025GH001434

**Published:** 2025-12-02

**Authors:** Vitul Agarwal, Falk Feddersen, Elizabeth Brasseale, Jeff S. Bowman, Uwe Send, Matthias Lankhorst, Sarah N. Giddings, Matthew Spydell, Xiaodong Wu, Ganesh Gopalakrishnan, Jeff Sevadjian, Katherine E. Berman, Shelby Marhoefer‐Jess, Andrew D. Barton

**Affiliations:** ^1^ Scripps Institution of Oceanography UC San Diego La Jolla CA USA; ^2^ School of Marine and Environmental Affairs University of Washington Seattle WA USA; ^3^ School of Oceanography Shanghai Jiao Tong University Shanghai China; ^4^ Department of Ecology, Behavior and Evolution UC San Diego La Jolla CA USA

**Keywords:** ROMS, norovirus, wastewater pollution, risk assessment, state‐space models, public health risk

## Abstract

Untreated wastewater enters the ocean at an outfall in Mexico and spreads to the San Diego‐Tijuana (USA‐Mexico) border region, posing significant risks to human health. Here, we developed a risk assessment tool for coastal communities, leveraging hindcast oceanographic simulations (2017–2019), to link changes in temperature and salinity at the coastline to high wastewater concentrations. We first calculated the modeled timescales (i.e., duration and return time) of wastewater exposure for popular beaches in the region. Most high wastewater exposure events occurred about once a month and lasted less than a week at the southern locations (e.g., Imperial Beach), and occurred less frequently and for shorter periods of time further north (e.g., Coronado). Using the same hindcast simulations, we then identified relationships between anomalous environmental conditions and wastewater concentration along the coastline. High wastewater concentrations were typically associated with lower salinity and temperature, reflecting the low salinity of wastewater and the colder temperatures of water originating south of the USA‐Mexico border. Statistical models with only parameters of salinity and temperature anomalies captured a large proportion of the variation in wastewater‐associated risk of illness (*R* ^2^ = 0.63–0.78). We tested the risk assessment approach with several months of recent observations (January–December 2024) to show how this tool may be practically applied. This study provides an efficient method for developing risk models that utilize commonly measured environmental data, with applications to other pollution‐impacted coastal locations.

## Plain Language Summary

Untreated wastewater released 10 km south of the USA‐Mexico border often reaches coastal areas in southern San Diego County, USA, and Tijuana, Mexico, posing health risks to humans. This study uses an ocean model to examine how often and how long these pollution events last, identify associations between environmental variables and risk of illness, and develop a pathogen risk assessment tool based on observations of water temperature and salinity. This work emphasizes the importance of regular ocean monitoring and the potential impacts of persistent water pollution on coastal communities.

## Key Points

- A hindcast ocean model (2017–2019) estimated that sewage pollution reaches coastal beaches in the US‐MX border region at least once a month
- Model nearshore water temperature and salinity anomalies were associated with wastewater contamination and the risk of illness
- Estimated risk, tested on 7 months of observations in 2024, moderately correlated (r=0.31) to shoreline *Enterococcus* concentration

## 1 Introduction

Wastewater pollution is a global problem, with an estimated 48% of all production entering the environment untreated (Jones et al., 2021). Of this large volume, coastal sewage pollution is of significant concern across many regions (Rangel‐Buitrago et al., 2024), with reported consequences ranging from the transmission of deadly human pathogens (Bogler et al., 2020; Piarroux et al., 2011), the fecal contamination of shellfish (Antony et al., 2021; Campos et al., 2015; Nagarajan et al., 2022), the degradation of coral reefs (Abaya et al., 2018; Lachs et al., 2019; Reopanichkul et al., 2009) or the detection of illicit drugs in marine life (de Farias Araujo et al., 2024). Chronic exposure to untreated wastewater in coastal waters may increase the risk of respiratory, gastrointestinal, and ocular infections, particularly for immunosuppressed or immunocompromised persons (Griffin et al., 2003).

In the past few decades, the flow of untreated wastewater from Mexico to the United States‐Mexico (US‐MX) border region in Southern California, has become a socio‐economic, political, environmental and human health issue (Allsing et al., 2023; ARCADIS, 2019; Ayad et al., 2020; Feddersen et al., 2021; McLamb et al., 2024). According to some estimates, millions of gallons of untreated sewage flow into the ocean per day, approximately 10 km south of the border, which is transported via ocean currents to coastal communities in San Diego and Tijuana every year (ARCADIS, 2019). Regular monitoring of human fecal indicator bacteria has consistently revealed unsafe conditions for recreational swimmers (Gersberg et al., 2006; Zimmer‐Faust et al., 2023) and led to near‐permanent beach closures in many areas. The “sewage crisis” has also prompted calls for significant investment into the monitoring and mitigation of wastewater that enters the San Diego‐Tijuana (US‐MX) border region.

Several recent studies have attempted to identify and track the pollutants present within wastewater in the region, including fluorescent organic matter (Mladenov et al., 2024), microbial pathogens (Allsing et al., 2023), and organic pollutants (McLamb et al., 2024). Other research has concentrated on the physical transport of pathogens using high resolution physical transport models to predict pathogen exposure along the shoreline (Feddersen et al., 2021; Wu et al., 2020) or by estimating exposure to contaminated sea spray aerosol (Pendergraft et al., 2023). A major limitation of these methods is that they either depend on regular field sampling of relevant parameters or involve computationally intensive simulations within a specified forecast period, which limits their flexibility in responding to rapidly changing hourly conditions.

A potential solution, as demonstrated by Brasseale et al. (2023), is to develop forecasts based on a one‐dimensional wave‐driven advection and loss model, which can successfully reproduce the performance of a three‐dimensional regional hydrodynamic model (Brasseale et al., 2023) at a lower computational cost. This can be extended to leverage the empirical relationship between relatively easily measured environmental parameters, like salinity or water temperature, to sewage contamination along the coastline. As human households consume large volumes of freshwater, including for wastewater‐generating activities such as doing laundry, showering, toilet flushing or dishwashing (Mazzoni et al., 2023; Roshan & Kumar, 2020), we posit that the low salinity of sewage‐contaminated seawater is a key indicator of wastewater contamination.

In this study, we develop an empirical risk assessment model that can provide real‐time information on water quality at beaches in San Diego, USA, and Tijuana, Mexico. We used hindcast oceanographic model simulations to identify the salinity and temperature conditions that are associated with high wastewater concentrations. We also used the model‐estimated wastewater fraction to characterize event duration and return time for several key coastal locations frequented by the local population. We then developed a metric that can empirically connect recent observations to the potential risk of illness from norovirus. We extended this approach built on historic data and simulations to recent observations and identified scenarios where risk assessment may be helpful to local communities. Overall, our work outlines the potential for wastewater risk assessment using in situ environmental observations in highly dynamic environments and may serve as a roadmap for other global sites that are currently experiencing significant challenges related to coastal pollution.

## 2 Materials and Methods

For a brief overview of how the methods relate to one another, please refer to an illustrative flow‐chart in Figure S1 in Supporting Information S1.

### 2.1 Hindcast Dynamic Simulations

The San Diego Bight model ($15\times 36\;km^{2}$) is a three‐dimensional regional coastal ocean model that two‐way couples the Regional Ocean Modeling System (ROMS) (Shchepetkin & McWilliams, 2005) with the Simulating WAves Nearshore (SWAN) model (Booij et al., 1999) using the Coupled Ocean‐Atmosphere‐Wave‐Sediment‐Transport (COAWST) modeling system (Kumar et al., 2012; Warner et al., 2010), to describe the physics and transport pathways for untreated wastewater across the US‐MX border region. The model introduces estimated volumes and concentrations of wastewater at known coastal locations where untreated wastewater enters the ocean (described below). Ocean circulation driven by realistic historical forcing (open ocean conditions, tides, waves, and atmospheric forcing) transports and dilutes the modeled wastewater. This model has been extensively used to study the physical dynamics of wastewater flow and illness risk in the San Diego—Tijuana border region (Feddersen et al., 2021). Wastewater was modeled as a dye with a decay constant consistent with that of norovirus; however, we do not perform norovirus concentration calculations and instead look at the wastewater fraction itself (Feddersen et al., 2021). Detailed model descriptions and applications can be found in previous studies (Feddersen et al., 2021; Wu et al., 2020, 2021a, 2021b). We used hindcast model output (2017–2019) to generate an hourly time series of salinity (PSU), temperature (°C) and fraction of wastewater concentration (D; ranges from 0 to 1, where 1 implies 100% wastewater) at several locations (described in Section 2.2). The use of hindcast model simulations allowed us to identify potential relationships between the three variables and create a risk assessment tool that can then be tuned to current observations. In general, the hindcast model output compares favorably to observations (e.g., Wu et al., 2020) and is significantly correlated to long‐term observations of environmental properties in the study region (Figure S2 in Supporting Information S1, *r* = 0.49, 0.93 for *S* and *T*).

### 2.2 Locations

**Figure 1:**
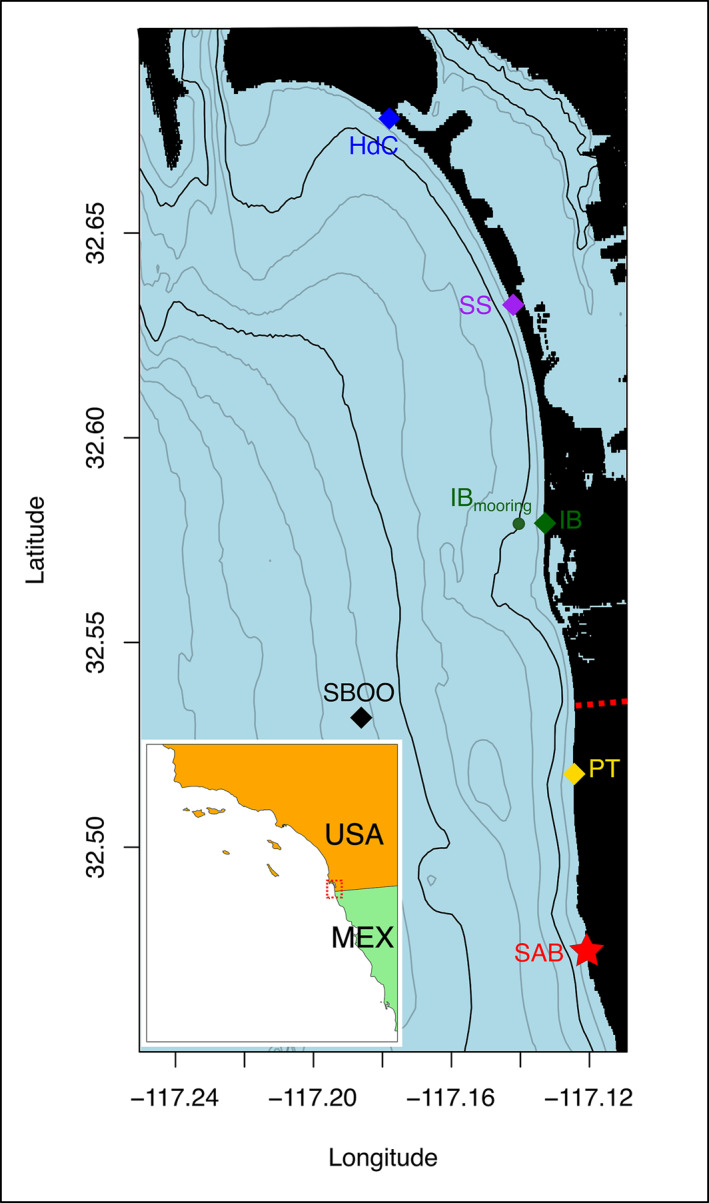
San Diego Bight (US‐MX border region) as a function of latitude and longitude spanning Point Loma to south of Punta Bandera, Mexico. Note that the full SD Bight model domain extends slightly beyond this figure as shown in Feddersen et al. (2021). The inset map shows the approximate location of the study area. The untreated wastewater source at the San Antonio de los Buenos outfall (SAB) located at the shoreline of Punta Bandera is indicated as a red star. Colored diamonds indicate beach locations at Playas Tijuana (PT; gold), Imperial Beach (IB; green), Silver Strand State Beach (SS; purple), and Hotel del Coronado (HdC; blue). Black diamond indicates the offshore South Bay Ocean Outfall location (SBOO). Small green circle is the location of the observational mooring at IB. *Enterococcus* data were collected less than 20 m from the IB location. Bathymetry contours every 5 m are shown in black lines (10 and 25 m depth contours in a darker line). The red dashed line indicates the US‐MX border.

This study focuses on a single point source of sewage, the San Antonio de los Buenos (SAB) treatment plant at Punta Bandera (PTB) in Mexico, approximately 10 km south from the US‐MX border (Figure 1). Untreated wastewater is directly ejected onto the beach (∼35 million gallons per day), which is transported and mixed northward along the shoreline to United States coastal waters. A secondary source of sewage, the Tijuana River Estuary (TJRE), is not considered in this study due to significant recent changes in the concentration of untreated sewage dispersal due to infrastructure failure. Over the period of model simulations (2017–2019), the largest source of wastewater into the coastal ocean was shown to be from PTB, and consequently we focus on that source in this study. Modeled TJRE sewage flow was low over the same period and had minimal environmental contributions. By ignoring the sewage contribution of TJRE, we avoid deriving and describing environment‐wastewater relationships that might have changed since 2019. Our study aims to capture the general physical conditions of an environment contaminated with wastewater and is necessarily linked only to the simulated dynamics of physical transport and dilution within coastal waters (i.e., there are no explicit biological processes).

We selected five specific locations along the US‐MX border region for conducting more detailed analyses (Table 1; Figure 1). Surface time series of salinity (PSU), temperature (°C) and wastewater concentration (D) were extracted for each of these locations from the SD Bight Model hindcast simulations. The South Bay Ocean Outfall (SBOO) was considered as the reference offshore location, whereas Imperial Beach (IB) was considered the primary coastal location of interest. SBOO is located approximately 7 km from the shoreline and tends to experience physical conditions with minimal influence from SAB sewage outflow. It is part of an existing environmental observational network that provides real‐time estimates of physical observations on an hourly time scale. Similarly, IB is a popular recreational beach and is regularly monitored for physical conditions and water quality. Playas Tijuana (PT), Silver Strand (SS) and Hotel del Coronado (HdC) are other coastal sites along the shoreline. These sites were selected for their importance to local communities and to develop a nuanced understanding of sewage risk during coastal plume events. The shoreline locations were extracted as done in a prior study (Feddersen et al., 2021).

**Table 1:** List of Locations of Interest and Their Coordinates

Location	Description	Latitude	Longitude	Water depth (m)
SBOO	South Bay Ocean Outfall	32.53163	−117.1861	∼30 m
PT	Playas Tijuana	32.51789	−117.1244	Shoreline
IB	Imperial Beach	32.57913	−117.1329	Shoreline
SS	Silver Strand State Park	32.63246	−117.1421	Shoreline
HdC	Hotel del Coronado	32.67796	−117.1780	Shoreline
IB_mooring_	Mooring (T and S obs.)	32.57924	−117.1418	∼10 m
EH‐030	*Enterococcus* obs.	32.57927	−117.1330	Shoreline

### 2.3 Estimation of Plume Duration and Return

Based on the hindcast model wastewater fraction time series for each location, we identified how frequently coastal pollution plume events were present at different points along the shoreline and how long they persisted at each location. We first converted the time series of wastewater concentration (log_10_ *D*) into a binary time series based on thresholds of high illness risk. Different wastewater concentrations imply different levels of illness risk to swimmers (Boehm & Soller, 2020). For this section of our analysis, we selected thresholds of $\log_{10}\;D=-4$ and $\log_{10}\;D=-3$ to focus on events that pose the greatest risk to local communities, estimated from the EPA recreational water quality illness probability threshold (Feddersen et al., 2021). Higher concentration of wastewater implies higher probability of illness. Each “event” is defined as all continuous sequences of time where wastewater concentrations were greater than the specified threshold. Thus, “event duration” was calculated as the length of time sustained above the threshold and “return time” was calculated as the time between events. For both calculations, only differences greater than 1 hr (the temporal resolution of the hindcast model output) were considered. We then calculated and reported the 90th percentile of the distribution for both event duration and return time. The 90th percentile was chosen instead of the median or mean value to highlight the timescales below which “most” events lie, which accounts for greater‐than‐average events while still excluding extreme outliers.

### 2.4 Risk Assessment

**Figure 2:**
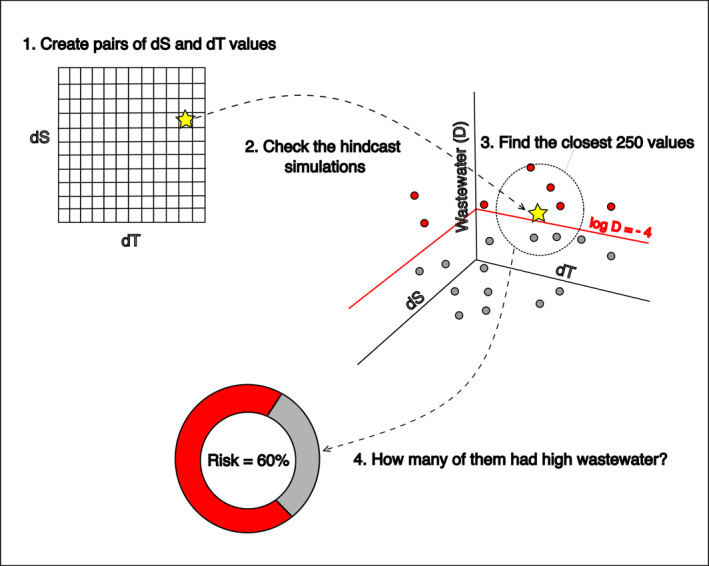
Illustration of the risk assessment calculation based on the state‐space method. For each pair of Δ*S* and Δ*T* (yellow star) at a given location, risk is reported as the proportion of the 250 nearest neighbors (red points within the dashed circle) with wastewater concentration $\log_{10}\;D>-4$.

We implemented a state‐space approach on the extracted hindcast model time series to identify the relationship between salinity, temperature and wastewater concentration (Figure 2). In other words, for a given environmental state (“Δ*S*” and “Δ*T*”) along the shoreline, how likely were high wastewater concentrations? To effectively isolate the influence of wastewater at the shoreline, as opposed to seasonal or oceanographic changes in model temperature and salinity, we first subtracted the offshore model salinity and water temperature at SBOO from the model salinity and water temperature at the shoreline locations (HdC, SS, IB and PT). The resulting model anomaly time series (“Δ*S*” and “Δ*T*”) and associated wastewater concentrations at each shoreline location were then used as a library for developing risk assessments.

To calculate risk as a function of temperature and salinity, we created two sequences of model Δ*S* (intervals of 0.01) and Δ*T* (intervals of 0.1). Then, for every pair of these sequences (Δ*S*, Δ*T*), we calculated the Euclidean distance of that pair of values from the entire library of Δ*S* and Δ*T* points (i.e., from the hindcast model output). We then identified the 250 nearest neighbors from the library and estimated the risk of illness as the proportion of those neighbors that were associated with wastewater concentrations exceeding the threshold $\log_{10}\;D=-4$. Risk was only calculated for a pair of Δ*S* and Δ*T* values if there were at least 250 points in the libraries within a Euclidean distance of 0.5. This limit was chosen to remove spurious state‐space associations and ensure that all risk calculations are comparable. The resulting estimated risk was reported as a % risk of illness, where 0% indicates that none of the nearest neighbors had wastewater concentrations greater than the threshold and 100% indicates that every nearest neighbor had wastewater concentrations greater than the threshold. The number of nearest neighbors and the threshold of concern determine the value and distribution of risk, so we chose 250 and $\log_{10}\;D=-4$ after conducting a sensitivity analysis (Figures S3 and S4 in Supporting Information S1). A greater number of nearest neighbors or a higher threshold results in lower estimated risk. Figure 2 illustrates the risk assessment calculation. This threshold ($\log_{10}\;D=-4$) is a wastewater fraction beyond which the estimated probability of swimmer illness (Feddersen et al., 2021) exceeds EPA recreational water quality illness probability thresholds. As such, the risk of illness metric in this study represents several related ideas: the likelihood of beach closures, the chances a swimmer will get sick from norovirus, and whether the environmental conditions suggest wastewater contamination.

### 2.5 Polynomial Regression Models

As additional support for the state‐space approach of estimating risk, we created a series of polynomial regression models to predict the estimated risk from the modeled Δ*S* and Δ*T*. In other words, we created equations for the relationships identified between Δ*S*, Δ*T*, and estimated risk from the state‐space approach (see Section 2.4; Figure 2). This was done to highlight the relative importance of both Δ*S* and Δ*T* in the estimation of risk. For each location, five separate models were created with varying parameters (Δ*S*, Δ*T*, Δ*S* ^2^, Δ*T* ^2^) with interactions between them. The first two models tested the performance of single‐parameter predictions, based solely on Δ*S* or Δ*T*, whereas the third model included the interaction term Δ*S*Δ*T*. The last two models introduced squared Δ*S* ^2^ and Δ*T* ^2^ parameters to test the potential of nonlinearity within the system. Regression model performance was reported as an adjusted *R* ^2^ value and an Akaike Information Criterion (AIC) score. The polynomial regression models were not used in subsequent analyses: they solely provide an alternative estimation method for situations where hindcast model simulations are unavailable.

### 2.6 Observational Data and Validation

To test the risk assessment approach (as described in Section 2.4), we implemented the state‐space calculation on recent salinity and temperature observations. We used salinity and temperature data from two mooring systems with the same near‐surface conductivity‐temperature‐depth sensors (SeaBird Electronics SBE‐37 at 1 m depth)—one at SBOO and the other near IB (32.579240, −117.141790), approximately 0.4 km beyond the end of the IB pier in 10 m water depth, from 1 January 2024, to 1 January 2025. The sensors had poison antifoulant plugs, provided by the manufacturer, at the intake and exhaust of the conductivity cells. A copper mesh was placed between the conductivity cell and guard, and copper tape was applied around the length of the housing. The instruments were calibrated by the manufacturer prior to deployment. In addition, bottle samples were collected at the same depths 1 m of the near‐surface instruments at the beginning, end, and periodically throughout each deployment, and analyzed for salinity. With these data, we created a Δ*S* and Δ*T* time series by matching up the sampling dates (numerically averaged to daily resolution) subtracting the temperature and salinity at SBOO from the IB time series. Risk was then calculated from our existing library of hindcast model simulations with the state‐space method.

Due to potential error in salinity measurements because of persistent biofouling, we devised an error‐estimation scheme to improve the robustness of risk assessments. Potential error was estimated based on concurrent bottle measurements (Supporting Information S1). For each value of Δ*S*, 1,000 random samples of a Gaussian distribution (Mean=−0.04;σ=0.04) were taken and added to the Δ*S* value. This distribution matched the typical difference and deviation of the bottle measurements from the mooring system. Consequently, each observational measurement had a distribution of 1,000 values of estimated risk. The mean estimated risk was reported as part of the analysis.

We compared the time series of state‐space estimated risk from these observations to daily‐resolution *Enterococcus* ddPCR data collected at the IB Pier shoreline location (California State Water Resources Control Board; https://www.waterboards.ca.gov/; EH‐030) in order to test the efficacy of the metric in tracking wastewater. The model extracted IB shoreline location is <20 m from EH‐030. *Enterococcus* is a proxy for wastewater contamination as it is widely used as a fecal indicator (Boehm & Sassoubre, 2014; Wheeler et al., 2002). Risk assessment performance is reported as the Pearson correlation coefficient between the time series of state‐space estimated risk using the SBOO and IB mooring observations and the observed ddPCR values of *Enterococcus* at the IB Pier location.

### 2.7 Software

Data analysis was conducted in R (R Core Team, 2024). The packages used were “ncdf4” (Pierce, 2023), “raster” (Hijman, 2023), “ggplot2” (Wickham, 2016), “cowplot” (Wilke, 2020), “maps” (Becker et al., 2022), “MASS” (Venables & Ripley, 2002), and “sf” (Pebesma, 2018). These packages were used for data handling, plotting, and enhancing the visual quality of the figures.

## 3 Results

### 3.1 Hindcast Model Simulations

**Figure 3:**
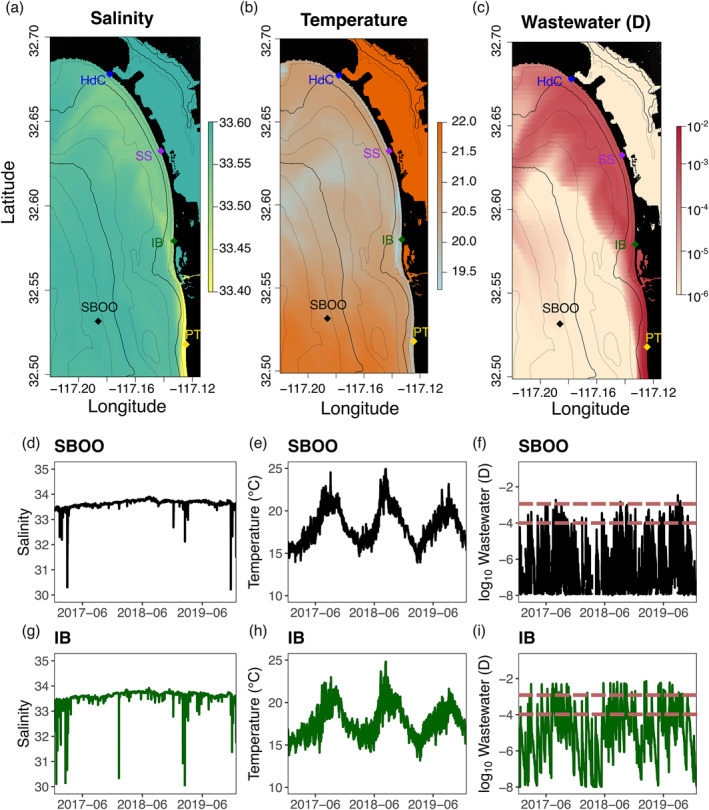
Hindcast simulation water quality output. The maps represent a single snapshot of model output on 2017‐07‐11 13:59:59 UTC: salinity (top left), temperature (top middle) and wastewater concentration (log_10_ *D*; top right). The bottom panels show the salinity, water temperature and wastewater concentration (log_10_ *D*) time series for two locations: South Bay Ocean Outfall (SBOO; black) and Imperial Beach shoreline (IB; dark green). The dashed lines on (f) and (i) are thresholds of $\log_{10}\;D=-4$ and $\log_{10}\;D=-3$. Note: color scales have been saturated (at both ends) to enhance the visual quality of the maps.

The hindcast ocean model simulated the three‐dimensional ocean circulation of the region and consequently established dynamic linkages between ocean environmental and wastewater conditions in the region (Figure 3). In several instances, sewage outflow spread northward from Mexico along most of the model domain coastline, from the source northward to Coronado Island (Figure 3c). In such cases, the role of wastewater dilution and mixing is apparent, with reduced concentrations and greater geographical spread as one moves further north. The corresponding maps for salinity (Figure 3a) and temperature (Figure 3b) also imply a relationship between the presence of wastewater and environmental properties. Particularly for the southernmost locations, such as PT or IB, wastewater concentration appears strongly associated with lower salinity and temperature. Over the entire hindcast period (2017–2019), the time series of salinity and water temperature exhibits highly dynamic behavior with integrated signals of seasonal cycles, sewage plumes, wind‐driven upwelling and tidal transport. The model predicted wastewater at both SBOO and IB, although the concentrations at IB are generally orders of magnitude greater than the concentrations at SBOO. Even though we disregarded TJRE wastewater inputs (i.e., the wastewater fraction D is only input at PTB), the hindcast model still includes the influence of the Tijuana River on temperature, salinity, and mixing in the local environment. Consequently, some of the very low salinity events in the time series (Figures 3d and 3g) correspond to periods with high river flow from the Tijuana River.

### 3.2 Plume Duration and Return Time

**Figure 4:**
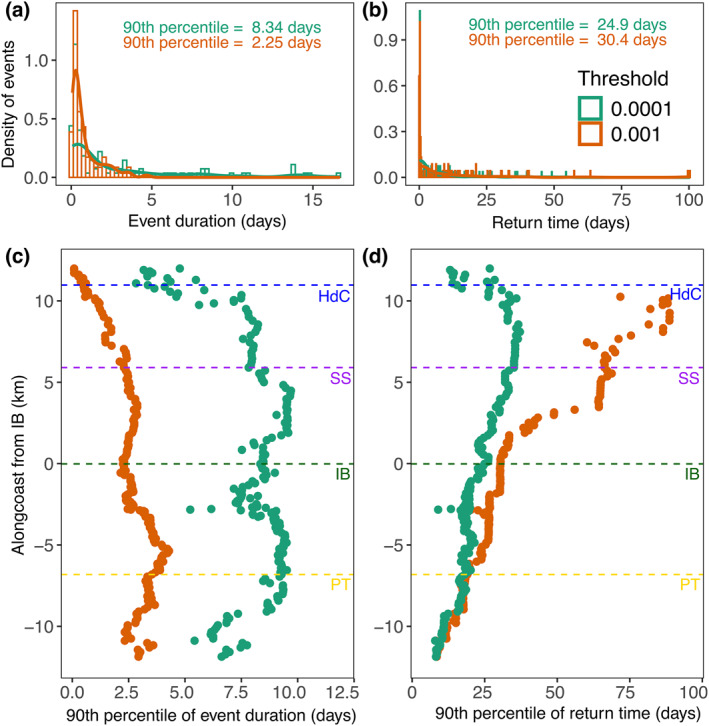
Duration and return time of sewage plumes. (a) The estimated duration (days) of sewage concentrations above the thresholds at IB. The bars represent the density of observations in 6‐hr intervals, and the solid lines are smoothed approximations of the distribution. The 90th percentile, in days, for both distributions is reported on the plot. (b) The estimated return time (days) of sewage concentrations above the thresholds at IB. The bars represent the density of observations in 6‐hr intervals, and the solid lines are smoothed approximations of the distribution. The 90th percentile, in days, for both distributions is reported on the plot. The 90th percentile of the alongshore estimated duration (c) and return time (d) for the entire shoreline in the hindcast model. The *y*‐axis has been converted to distance (km) from IB, which is represented by the green dashed line. For each panel, orange is for $\log_{10}\;D=-3$ threshold and teal is for $\log_{10}\;D=-4$ threshold. The colored dashed lines represent the other locations of interest along the shoreline. Outliers that exceeded 100 days were removed in panels (b) and (d).

Wastewater plumes at IB only last about a week, depending on the detection threshold (Figure 4a). For the higher threshold, signifying the worst wastewater events, the duration was about 2 days (Figure 4a; teal), extending to ∼8 days for the lower threshold (Figures 4a and 4b; orange). In other words, wastewater plume events caused by advection from Punta Bandera are short in duration, if intense. Interestingly, the plume event return time was consistent for both thresholds, suggesting that most events recurred about once a month at IB (Figure 4b). Across the entire shoreline, the duration of elevated sewage concentrations above the lower threshold was about 5–10 days at all locations except the northernmost sites (Figure 4c; teal), whereas the duration was less than 5 days for the higher threshold, with most values at or below 2.5 days (Figure 4c; orange). In contrast, the return time increased as one moved further north along the shoreline, with the sharpest increase for the events that exceeded the higher threshold (Figure 4d; orange). Return times started dropping again for the lower threshold around 8 km north of IB, reaching less than ∼25 days for HdC (Figure 4d; teal). In summary, locations further away from Punta Bandera experience shorter and less frequent wastewater exposure in most situations, the exception being some sites like HdC, where the frequency of low threshold events can match or exceed that of locations like IB.

### 3.3 Risk Assessment

**Figure 5:**
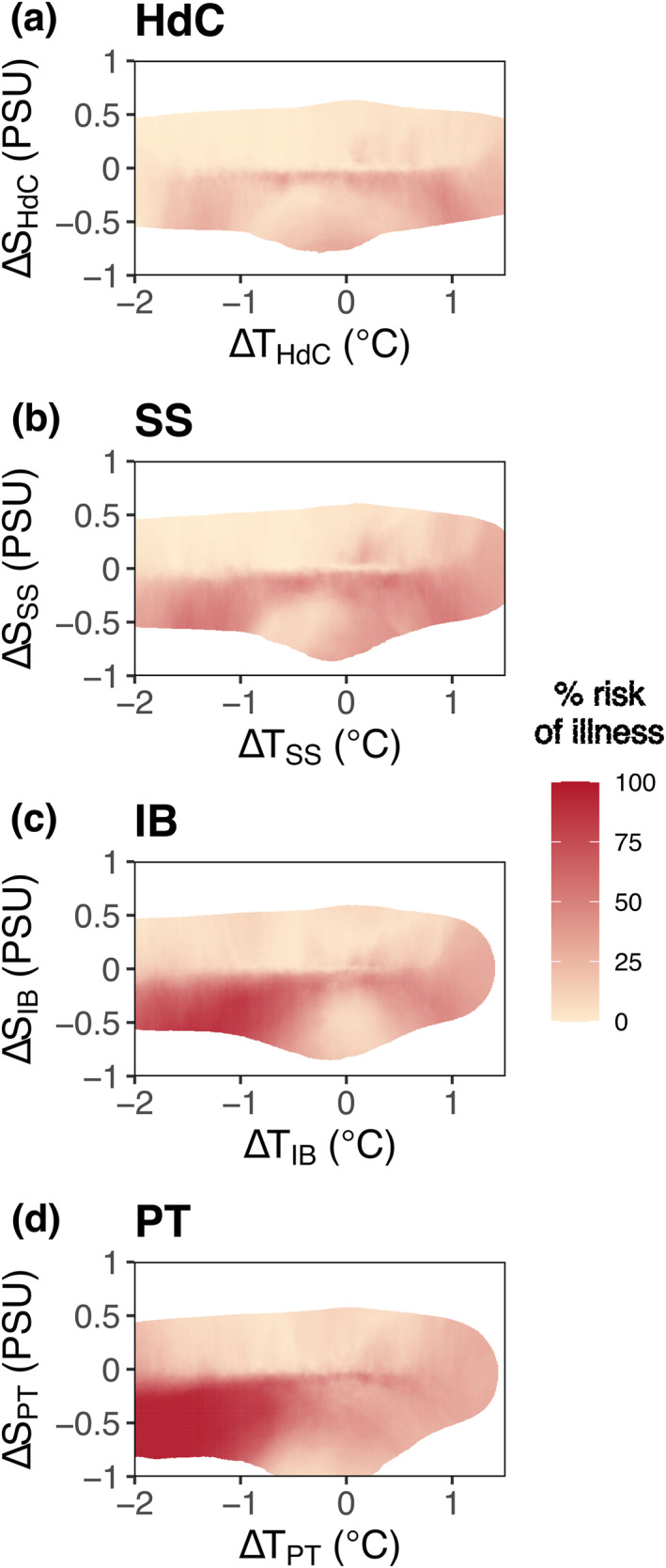
Estimated risk of illness (%) as a function of Δ*S* and Δ*T* for each coastal location (a) HdC, (b) SS, (c) IB and (d) PT. Δ*S* and Δ*T* is the arithmetic difference between the time series at those locations and the time series at SBOO (e.g., ${\Delta T}_{\text{HdC}}=T_{\text{SBOO}}-T_{\text{HdC}}$). Risk is calculated from a state‐space approach (see *Methods*) with a threshold of $\log_{10}\;D=-4\;\text{and}\;250$ nearest neighbors. White space indicates there were too few nearest neighbors within the library.

Risk at IB, defined from the state‐space method as the proportion of nearest neighbors with high wastewater fraction and calculated from a library of hindcast model simulations, had the highest values when Δ*S* and Δ*T* were both negative (Figure 5). For all positive Δ*S*, risk was near zero. Risk was also low when Δ*T* was around 0, across most Δ*S*. The patterns at other locations were similar to the pattern at IB (Figures 5a, 5b, and 5d). Relative to IB, values of risk were lower for the northernmost locations (Figures 5a and 5b; HdC and SS), and slightly higher for the southernmost location, PT (Figure 5d), closer to the Punta Bandera source. Overall, these results corroborate the expected relationship between salinity and wastewater, where a fresher signal (negative Δ*S*) was associated with higher risk of illness across all locations ($\log_{10}\;D>-4$). Cooler water temperatures at the shoreline (negative Δ*T*) were also associated with the risk of illness, particularly for the southernmost locations, PT and IB (Figures 5c and 5d). Increasing distance from the wastewater source at Punta Bandera also yielded lower risk, consistent with greater dilution and mixing over the transit distance/time.

**Figure 6:**
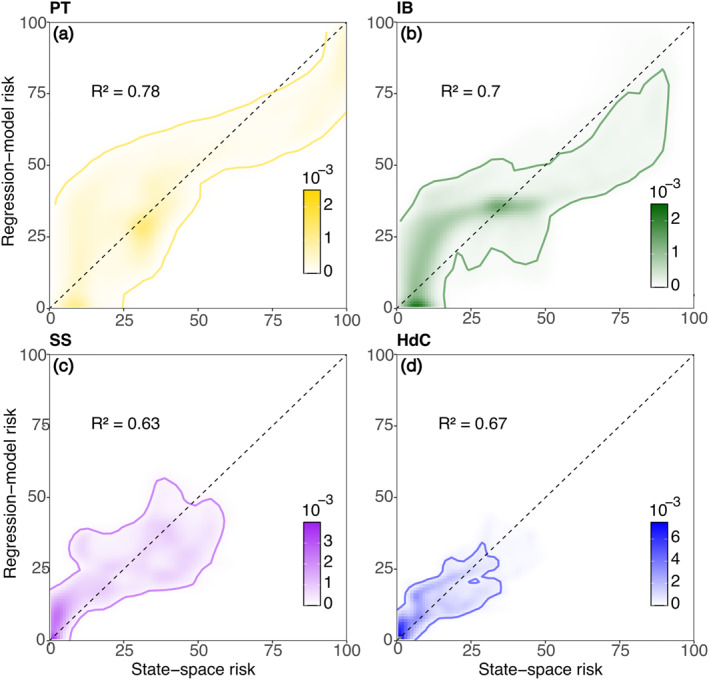
Regression risk models with just two independent variables (Δ*S* and Δ*T*) capture a large proportion of the variability in estimated state‐space risk for (a) PT, gold (b) IB, green (c) SS, purple and (d) HdC, blue. Shaded areas indicate the density of points. The solid contour lines represent 5% of the maximum density. Regression‐model risk is calculated from the best‐performing regression model (as listed in Table 2). State‐space risk is calculated from the hindcast simulations (as shown in Figure 5).

For each location, we developed a series of polynomial regression models to predict risk at each location based upon temperature and salinity information only (Figure 6). The more complex regression models (i.e., those that included squared parameters of Δ*S* or Δ*T*) outperformed the models with only linear Δ*S* and Δ*T*. AIC scores, which penalize models for overfitting, confirmed the increase in model skill for the more complex models (AIC − AIC_min_ = 0) relative to those with linear terms. Table 2 lists all the models we tested and their associated performance in describing the relationship between Δ*S*, Δ*T* and the risk of illness. As before (Figure 5), the highest risk was at the southernmost location, PT, decreasing northward (Figure 6; contour lines). The skill between the regression models and the state‐space risk models matched this trend, with better performance for PT (Figure 6a; $R^{2}=0.78$), followed by IB (Figure 6b; $R^{2}=0.70$), HdC (Figure 6d; $R^{2}=0.67$) and SS (Figure 6c; $R^{2}=0.63$). For the northern locations (HdC and SS), the model that performed best had squared Δ*T* terms (Figures 6c and 6d), whereas for the southern locations (IB and PT), the best models had squared Δ*S* terms (Figures 6a and 6b), implicating a greater effect of the freshwater signal for those locations (Figures 5c and 5d). Though idealized, these results indicate that even simple models including limited environmental information may provide skillful estimates of wastewater risk.

**Table 2:** For Each Location, List of Polynomial Regression Models, Skill (*R* ^2^), and AIC Score Relative to the Minimum AIC Score (AIC_min_) Across the Candidate Models

Location	Model	*R* ^2^	AIC − AIC_min_
PT	y=c+αΔS	0.31	103366.7
PT	y=c+βΔT	0.18	118670.9
PT	y=c+αΔS+βΔT+γΔSΔT	0.64	44051.6
PT	$y=c+\alpha \Delta S+\beta \Delta T+\gamma \Delta S\Delta T+\delta {\Delta S}^{2}+\varepsilon {\Delta S}^{2}\Delta T$	0.78	0
PT	$y=c+\alpha \Delta S+\beta \Delta T+\gamma \Delta S\Delta T+\delta \Delta T^{2}+\varepsilon {\Delta S\Delta T}^{2}$	0.70	29452.3
IB	y=c+αΔS	0.39	54426.1
IB	y=c+βΔT	0.04	89825.6
IB	y=c+αΔS+βΔT+γΔSΔT	0.59	23723.5
IB	$y=c+\alpha \Delta S+\beta \Delta T+\gamma \Delta S\Delta T+\delta {\Delta S}^{2}+\varepsilon {\Delta S}^{2}\Delta T$	0.70	0
IB	$y=c+\alpha \Delta S+\beta \Delta T+\gamma \Delta S\Delta T+\delta \Delta T^{2}+\varepsilon {\Delta S\Delta T}^{2}$	0.64	13913.8
SS	y=c+αΔS	0.44	32418.7
SS	y=c+βΔT	0.07	73294.7
SS	y=c+αΔS+βΔT+γΔSΔT	0.55	15824.6
SS	$y=c+\alpha \Delta S+\beta \Delta T+\gamma \Delta S\Delta T+\delta {\Delta S}^{2}+\varepsilon {\Delta S}^{2}\Delta T$	0.61	3526.8
SS	$y=c+\alpha \Delta S+\beta \Delta T+\gamma \Delta S\Delta T+\delta \Delta T^{2}+\varepsilon {\Delta S\Delta T}^{2}$	0.63	0
HdC	y=c+αΔS	0.43	44813.9
HdC	y=c+βΔT	0.16	76855.1
HdC	y=c+αΔS+βΔT+γΔSΔT	0.63	8822.4
HdC	$y=c+\alpha \Delta S+\beta \Delta T+\gamma \Delta S\Delta T+\delta {\Delta S}^{2}+\varepsilon {\Delta S}^{2}\Delta T$	0.64	7125.9
HdC	$y=c+\alpha \Delta S+\beta \Delta T+\gamma \Delta S\Delta T+\delta \Delta T^{2}+\varepsilon {\Delta S\Delta T}^{2}$	0.67	0

*Note*. y refers to the regression‐model risk of illness, which is predicted from ΔS and ΔT. c,α,β,γ,δ,andε were fit independently for each model.

### 3.4 Testing Against Concurrent *Enterococcus* Data

**Figure 7:**
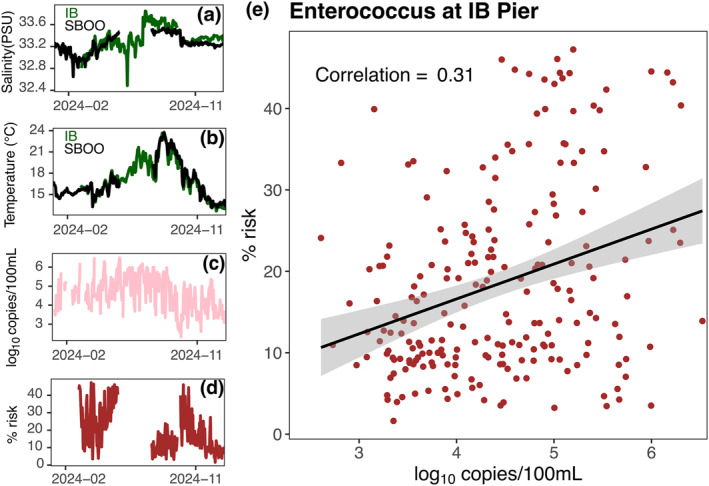
Application of hindcast model relationships to offshore observational data sets reveal differences in estimated risk and measured shoreline *Enterococcus* concentration at IB. (a) Offshore salinity measurements at IB (dark green) and SBOO (black), (b) Offshore temperature measurements at IB (dark green) and SBOO (black), (c) Shoreline *Enterococcus* concentration measured with ddPCR (log_10_ copies mL^−1^), (d) estimated risk from the state‐space method (%) and (e) Pearson correlation between measurements and risk assessment. The thick black line is a linear model that represents the relationship between risk and *Enterococcus* measurements, and the gray shading is the standard error of the linear model. For every time series, all available observations in the year 2024 were included in the analysis.

While the relationship between Δ*S* and Δ*T* and the risk of illness is clear in the hindcast simulations (Figures 5 and 6), we implemented the risk assessment approach for observational data collected by two offshore moorings at IB and SBOO for the year of 2024 (Figures 7a and 7b; Figure S5 in Supporting Information S1) to test the approach with in situ data. We then compared the risk estimate from these moorings with daily shoreline measurements of *Enterococcus* concentration (Figures 7c–7e). The calculated risk of illness varied from 0% to 50% (Figure 7d), with occasional gaps due to missing Δ*S* and Δ*T* measurements. The results highlight an interesting pattern—the historic oceanographic model library captured a small proportion of variance in observational data (Figure 7e; r=0.31). This means that simulated temperature, salinity, and wastewater relationships in the model can provide an estimate of risk that correlates with concurrent *Enterococcus* measurements. Conversely, error analysis indicated that there are several instances where the calculated risk failed to represent the *Enterococcus* concentrations in the environment, especially when the Δ*S* observations are positive (Figure S6 in Supporting Information S1). As will be discussed further below, it is important to remember that this observational comparison is not comparing measurements at the same location. The IB mooring from which Δ*S* and Δ*T* are calculated is located ∼0.8 km from the shoreline in ∼10 m water depth, whereas the *Enterococcus* measurements are measured at the shoreline within the surf zone.

## 4 Discussion

### 4.1 Timescales of Exposure to Sewage Plumes

The calculation of transport times, especially with the use of coastal ocean circulation models, is typically used for many reasons, such as understanding changes in water exchange due to coastal infrastructure (Gómez et al., 2014) and river discharge (de Pablo et al., 2022), or for estimating the age and arrival time of environmental contaminants (Shi & Yu, 2013). Here, the use of high‐resolution hindcast simulations allowed us to track and identify specific sewage plume events and calculate exposure timescales at different shoreline locations.

Even within a model with defined physical transport mechanisms, our estimates on event duration and return time suggest a landscape of complex behavior of wastewater transport and persistence. For example, different shoreline locations have distinct exposure patterns to wastewater (Figure 4). Locations such as IB experience less frequent, but longer‐lasting episodes of elevated wastewater, whereas others, such as HdC, experience more frequent and shorter episodes of elevated wastewater (Figures 4d and 4e). These patterns reflect a complicated interaction between the physical drivers and shoreline properties in the region, such as how wave advection interacts with near‐shore bathymetry (Brasseale et al., 2023), cross‐shelf exchange (Hally‐Rosendahl et al., 2014) the development of rip currents (Moulton et al., 2017), and the influence of the Tijuana River (Biggs et al., 2022; Gersberg et al., 2004).

This complexity may necessitate different strategies for public health management. For some locations such as IB, a strategy that identifies and predicts specific plume events may be more promising, as the events are less frequent but of longer duration. At others such as HdC, the increased frequency of low threshold and short duration events suggest public health management could focus on surveillance and mitigation (Groseclose & Buckeridge, 2017), especially for those regularly interacting with contaminated seawater.

### 4.2 Real‐Time Risk Assessment

Wastewater exposure risk, as estimated from the hindcast simulations, revealed a strong relationship between sewage and freshwater. For every location along the coast, higher risk was associated with fresher water (Figure 5). This implies that the salinity of sewage‐contaminated seawater may be a useful indicator for real‐time risk assessment, especially for shoreline locations closer to the source of sewage (Figures 6a and 6b; Table 2). Such a relationship is neither unexpected nor surprising, given that a large proportion of human wastewater will have a freshwater signal (Mazzoni et al., 2023; Roshan & Kumar, 2020).

In contrast, the association between high wastewater concentration and water temperature is less obvious but was picked up in both the hindcast simulations and the observational data set. Given that the point source of sewage is 10 km south of the US‐Mexico border, negative temperature anomalies are likely associated with upwelling along the Mexican coast (Jacox et al., 2018; Pickett & Paduan, 2003) where the wastewater is released. This suggests that released wastewater adopts the cooler signature of upwelled water and retains it for at least 12–18 km along the shoreline before the negative Δ*T* association seems to break down. An additional influence on water temperature is diurnal heating or cooling within the Tijuana River or at the SAB point source, leading to the discharge of sewage plumes with varying thermal characteristics depending on time of day.

When implemented on observational mooring data, the risk metric was positively correlated with the measurements of shoreline *Enterococcus* concentration (Figure 7e). The correlation (∼0.31) was not atypical, as prior studies that have estimated the risk of gastrointestinal illness in relation to wastewater exposure have reported widely‐varying estimates for marine environments (Wade et al., 2003). A possible explanation for the weak positive correlation is the distance between the shoreline (where *Enterococcus* was measured) and the mooring system (where temperature and salinity were measured). Even in the hindcast ocean circulation model, wastewater concentrations decreased with distance from the shoreline (Figure S7 in Supporting Information S1). For some wastewater exposure events, it is likely that our current environmental sampling system might be too far away from the shoreline to observe changes in the environmental parameters, or that the state‐space assessment needs to be adjusted for observed locations relative to the shoreline sampling locations.

Despite the hindcast model largely showing negative Δ*S* associated with wastewater (Figure 4), estimated risk for observations revealed many instances where wastewater was associated with positive Δ*S* (Figure 7a). While much of this could be attributed to sampling error, especially for salinity (Figure S2 in Supporting Information S1), or the distance of our observational system from the shoreline (Figure S7 in Supporting Information S1), it is also possible that there are differences in the environment since 2019 which were not captured in the hindcast simulations. For practical applications, establishing real‐time observational system closer to the shoreline and with sensors less prone to biofouling would increase performance and reliability of real‐time empirical risk assessment methods.

## 5 Conclusion

Persistent oceanic wastewater pollution along the San Diego‐Tijuana border region has led to beach closures and increased the potential risk of illness among swimmers and residents. By tracking hindcast ocean circulation model simulations of sewage outflow from the SAB treatment plant at Punta Bandera, we first identified the timescales of wastewater exposure (i.e., duration and return time) to potential swimmers. Shoreline locations far from the sewage source experience shorter, but more frequent, low‐threshold wastewater exposure events (northernmost sites; HdC) whereas locations closer to the source experience longer, but less frequent high‐threshold events (IB). These results provide a basis for the development of public health management strategies across the border region.

Second, we identified the relationship between anomalies in environmental parameters (temperature and salinity) and high wastewater concentration for popular beach destinations. These relationships were leveraged to develop a real‐time, empirical state‐space based risk metric that can provide an efficient assessment of water quality across the shoreline. The state‐space risk metric was also compared to polynomial regression models and then tested with observed offshore environmental parameters and shoreline bacterial count data. These results highlight the potential of observational data, trained with hindcast model simulation output, to predict wastewater at IB.

With further data collection by observational systems, real‐time risk assessment will be a useful complementary tool for water quality management in coastal San Diego (USA) and Tijuana (Mexico). Future risk assessments could rely entirely on observational moorings, integrating exposure risk with temperature and salinity data in public dashboards.

## Conflict of Interest

The authors declare no conflicts of interest relevant to this study.

## Supporting information

Supporting Information S1

## Data Availability

Time series of salinity, temperature, and wastewater concentration for all five primary locations of interest are available at Zenodo alongside code to reproduce the main figures in the manuscript (Agarwal, 2025). Observational data can be found at mooring.ucsd.edu.
