# Supplementary material for: Real‐Time Empirical Risk Assessment From Recurrent Coastal Sewage Plumes
Source: Geohealth. 2025 Dec 2;9(12):e2025GH001434. doi: 10.1029/2025GH001434 (PMC12672925; doi:10.1029/2025GH001434)
Supplement: Supplementary file 1 — Supporting Information S1 [file GH2-9-e2025GH001434-s001.pdf]

GeoHealth

Supporting Information for

Real-time empirical risk assessment from recurrent coastal sewage plumes

Vitul Agarwal[1*], Falk Feddersen[1], Elizabeth Brasseale[2], Jeff S. Bowman[1], Uwe Send[1], Matthias Lankhorst[1], Sarah N. Giddings[1], Matthew Spydell[1], Xiaodong Wu[3], Ganesh Gopalakrishnan[1], Jeff Sevadjian[1], Katherine E. Berman[1], Shelby Marhoefer-Jess[1], and Andrew D. Barton[1,4]

[1]Scripps Institution of Oceanography, UC San Diego, La Jolla, CA, USA
[2]School of Marine and Environmental Affairs, University of Washington, Seattle, WA, USA
[3]School of Oceanography, Shanghai Jiao Tong University, Shanghai, China
[4]Department of Ecology, Behavior and Evolution, UC San Diego, La Jolla, CA, USA

Contents of this file

Figures S1 to S7
Supplementary Data (Table S1)

Supplementary Figures

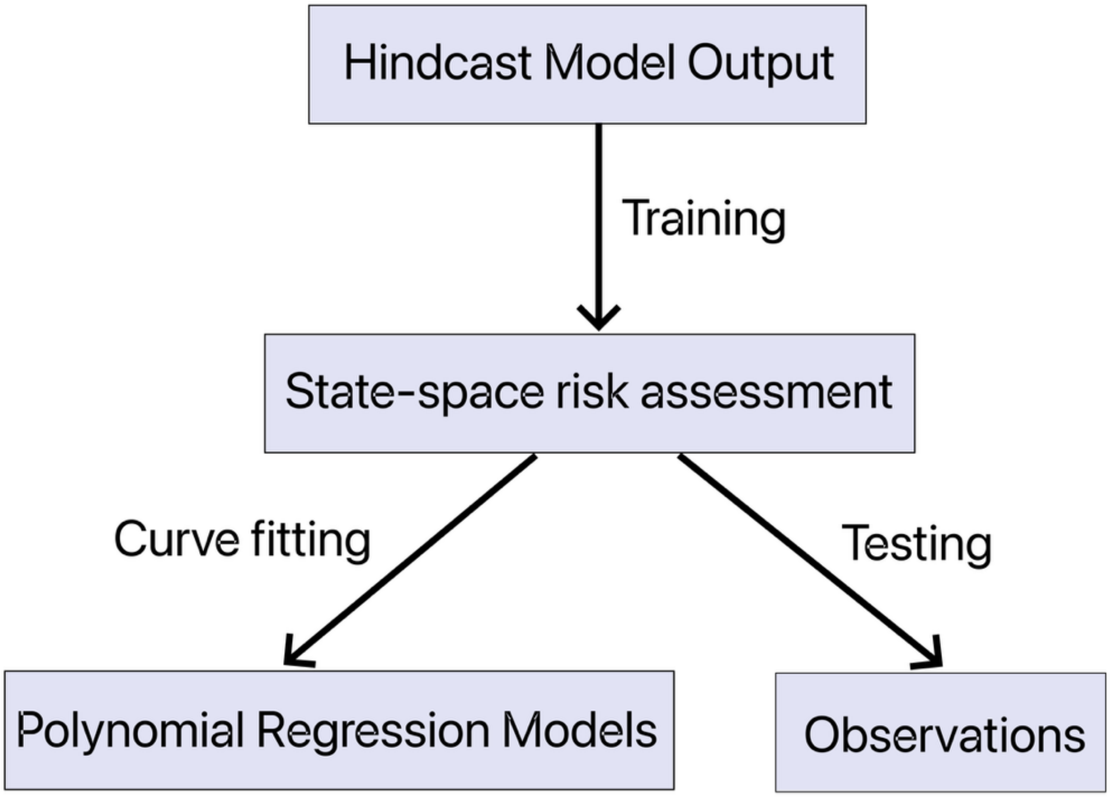

Figure S1: An illustrative flow-chart depicting the different methods and how they connect to one another.

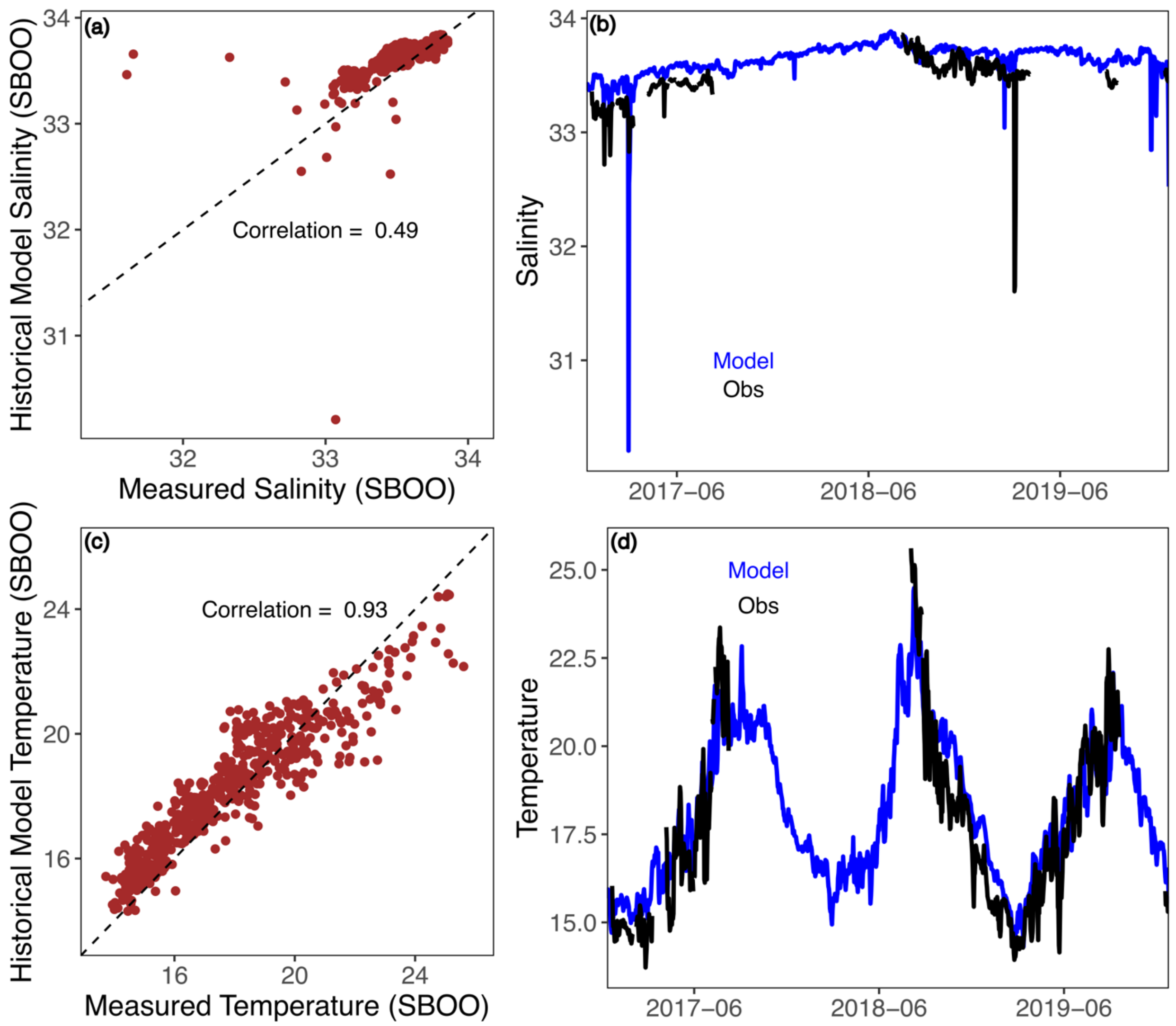

Figure S2: Salinity (PSU) and temperature (°C) values from the hindcast model simulations are correlated to long-term observations at SBOO. Blue is the model output and black is the observational time series. Note: There are fewer quality-controlled salinity measurements during 2017-2019.

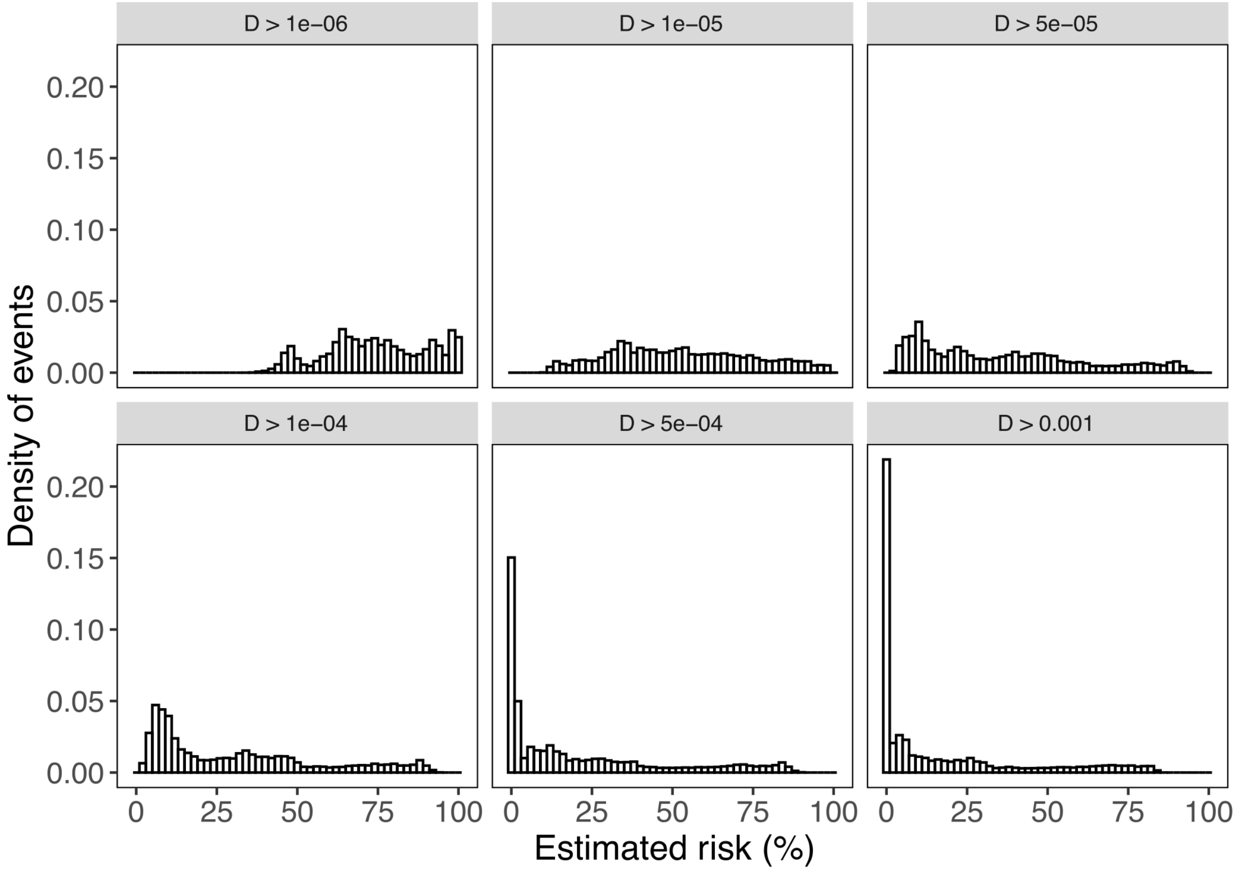

Figure S3: Estimated values of risk decrease with greater thresholds for events. Each panel shows the distribution of risk for the same dataset, with the only difference being the threshold used (panel title) to calculate risk.

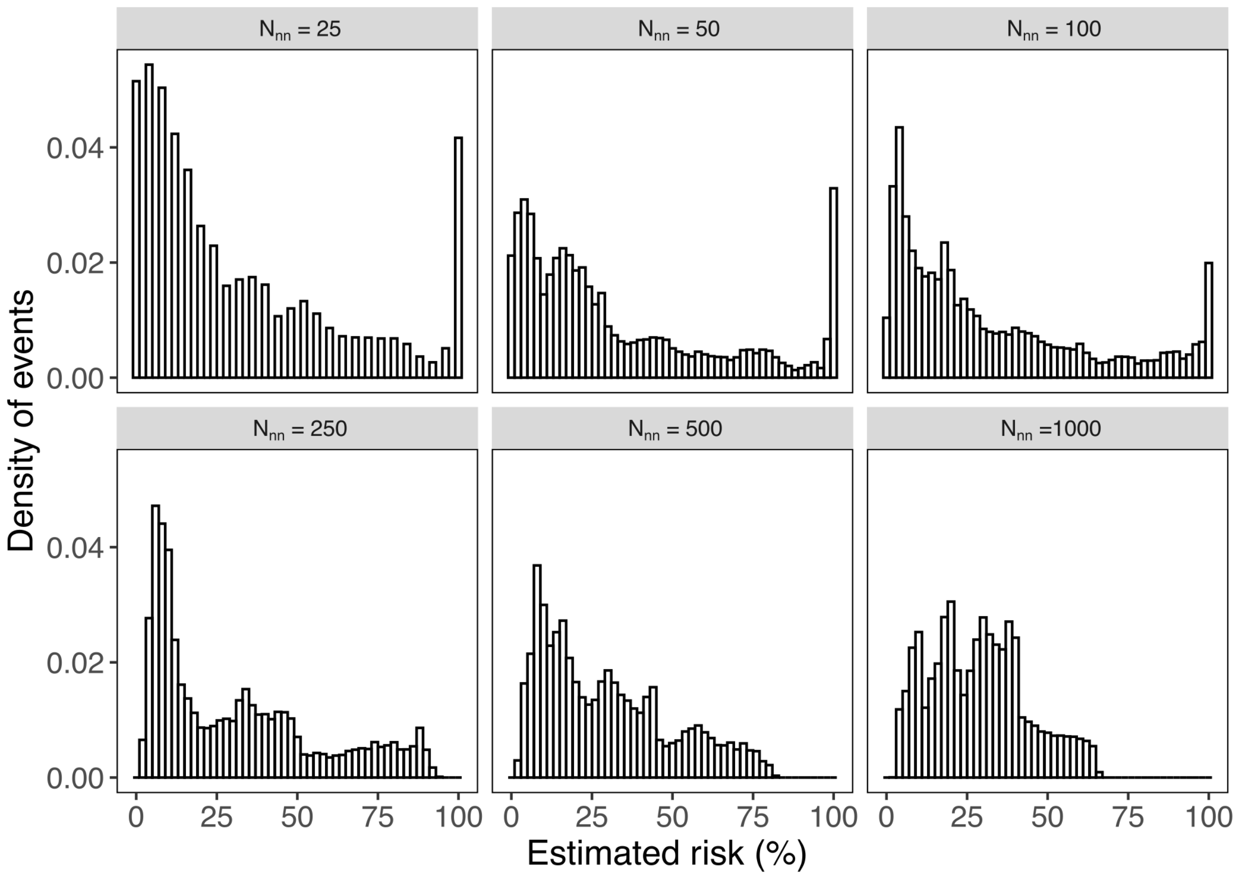

Figure S4: Estimated values of risk decrease with smaller numbers of nearest neighbors. Each panel shows the distribution of risk for the same dataset, with the only difference being the number of nearest neighbors used in the state-space calculation (panel title).

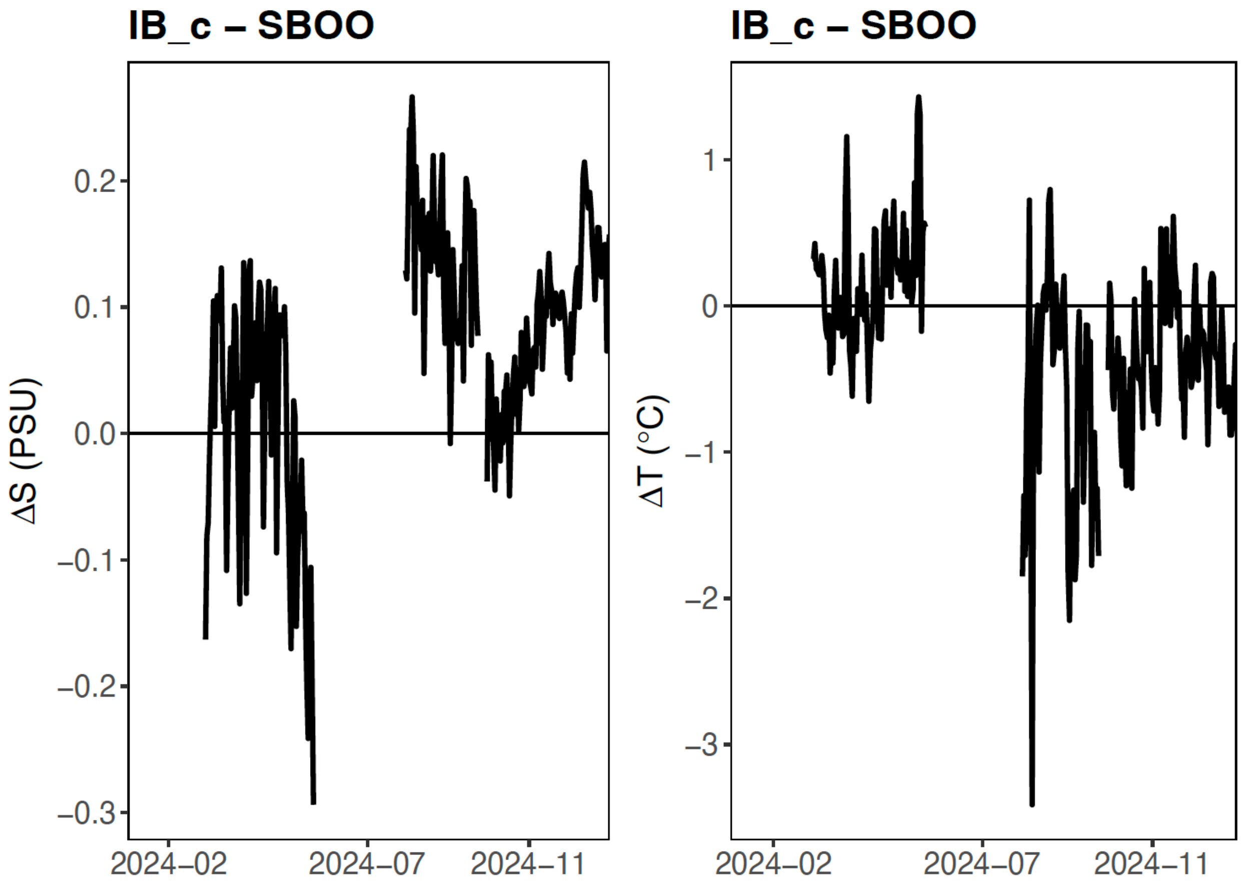

Figure S5: The difference in observational salinity (ΔS) and temperature (ΔT) measurements between IB and SBOO. For both time series, all available observations in the year 2024 were included in the analysis.

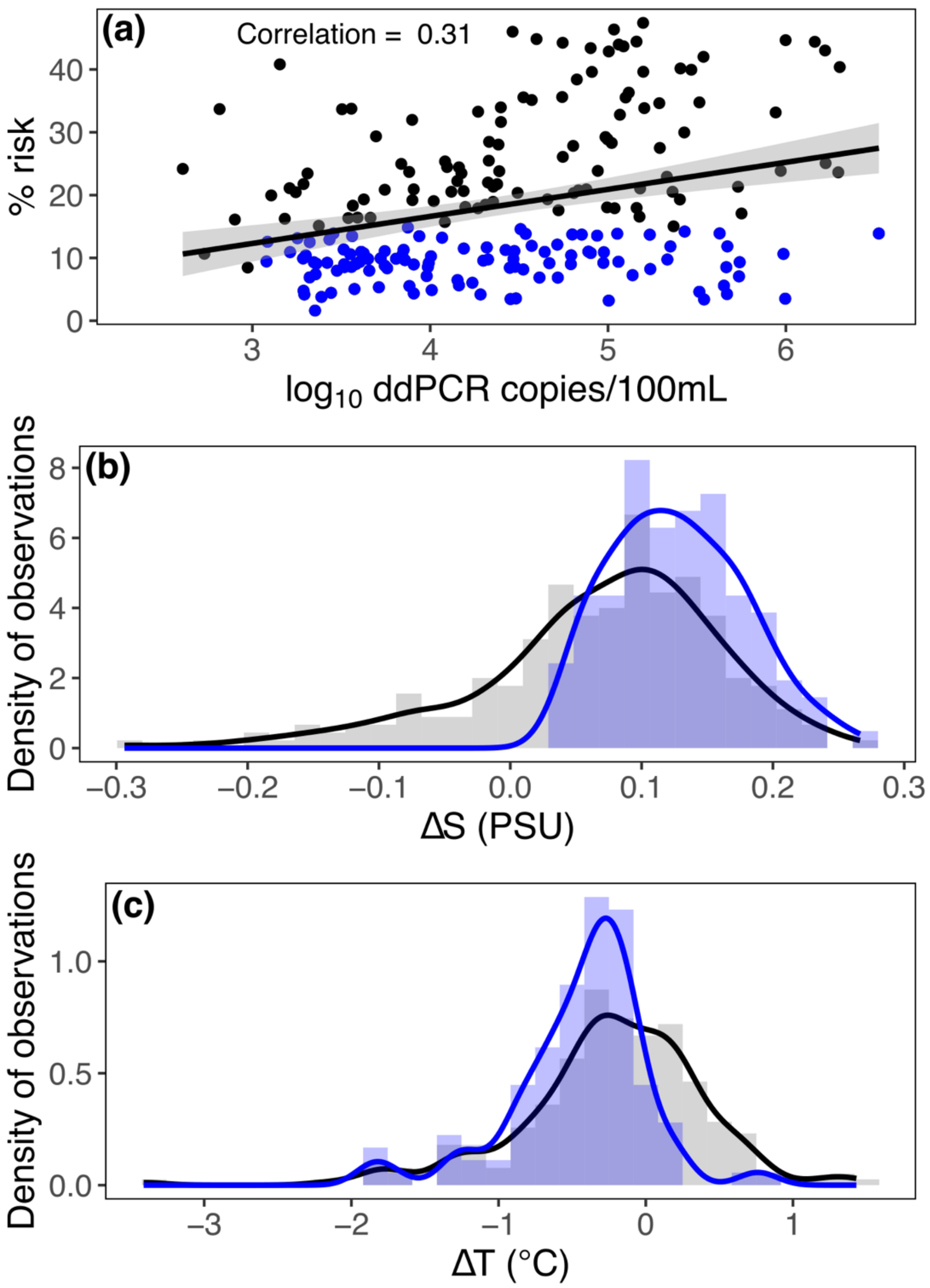

Figure S6: Risk assessment based on hindcast dynamic model simulations fails to capture many wastewater events, particularly when ΔS is positive and ΔT is negative. Blue points are all instances where the calculated risk was below 15% but the *Enterococcus* concentrations were greater than 1000 copies/100mL. (b) and (c) show the distribution of ΔS and ΔT values for the black and blue points in the top panel.

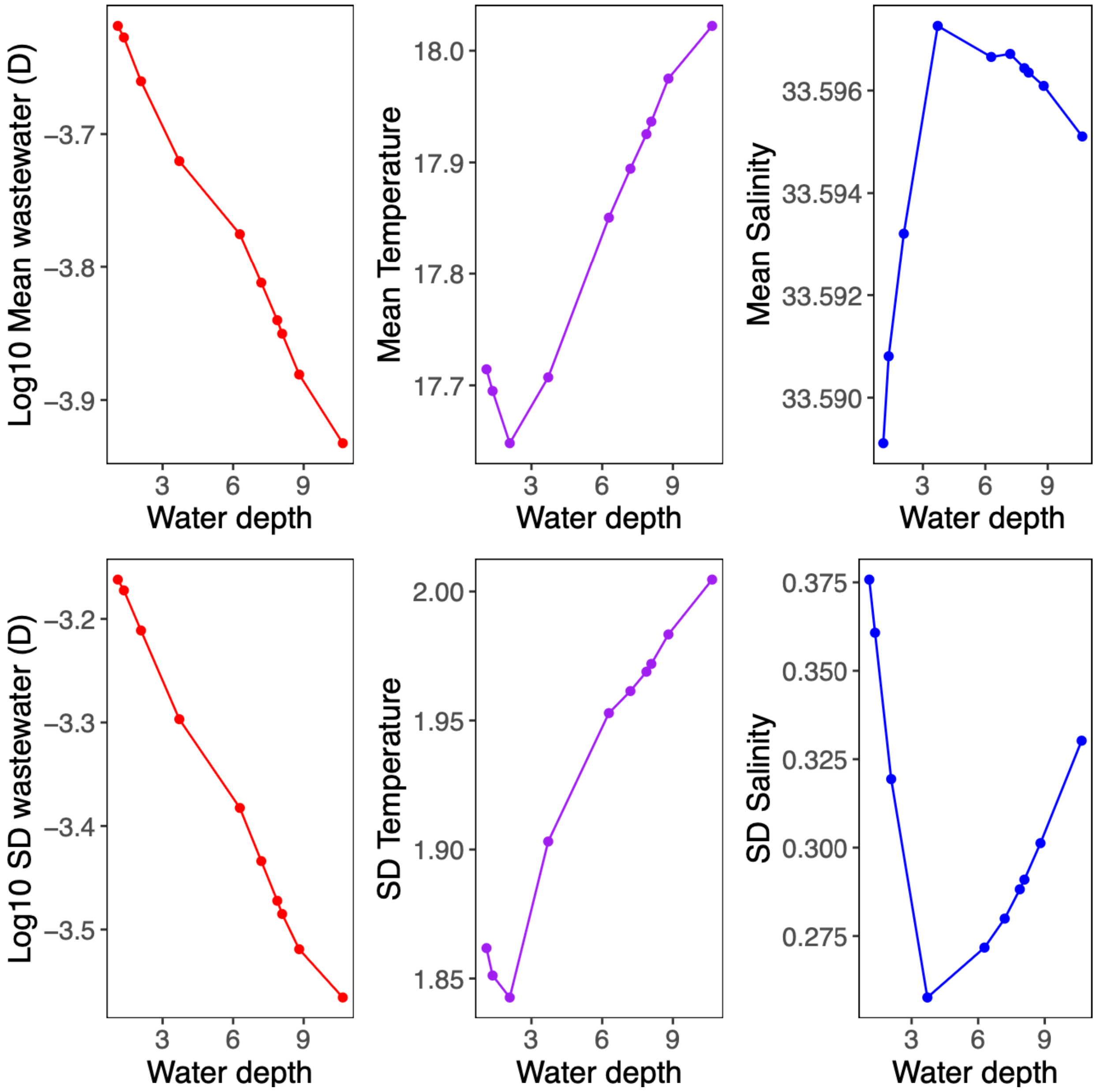

Figure S7: Hindcast model simulations at IB indicate that the wastewater concentration decreases as one moves away from the shoreline. Top row shows the mean for wastewater dilution (D; red), water temperature (°C; purple) and salinity (PSU; blue). Similarly, the bottom row shows the standard deviation. The observational mooring system is located at approximately 10m water depth.

Supplementary Data

Table S1: Bottle measurements of water salinity and how they compare to the mooring sensors.

Date	Time	Salinity (Bottle)	Salinity (Mooring)	Difference
**09/17/2024**	9:53	33.4646	33.494	-0.0294
**09/24/2024**	10:12	33.3154	33.391	-0.0756
**09/24/2024**	10:12	33.3155	33.391	-0.0755
**09/24/2024**	10:12	33.3179	33.391	-0.0731
**09/30/2024**	13:30	33.2414	33.226	0.0154
**09/30/2024**	13:30	33.2451	33.226	0.0191
**09/30/2024**	13:30	33.2413	33.226	0.0153
**10/01/2024**	7:38	33.222	33.313	-0.091
**10/01/2024**	7:38	33.2232	33.313	-0.0898
**10/01/2024**	7:38	33.2241	33.313	-0.0889
**10/15/2024**	7:36	33.2774	33.28	-0.0026
**10/15/2024**	7:36	33.256	33.28	-0.024
**10/15/2024**	7:36	33.2591	33.28	-0.0209
**11/1/2024**	7:57	33.2556	33.322	-0.0664
**11/1/2024**	7:57	33.2483	33.322	-0.0737
**11/1/2024**	7:57	33.2342	33.322	-0.0878
**11/06/2024**	8:20	33.2878	33.312	-0.0242
**11/06/2024**	8:20	33.2901	33.312	-0.0219
**11/06/2024**	8:20	33.2962	33.312	-0.0158
**11/19/2024**	8:19	33.3259	33.348	-0.0221
**11/19/2024**	8:19	33.3194	33.348	-0.0286
